# Primary care multidisciplinary teams in practice: a qualitative study

**DOI:** 10.1186/s12875-017-0701-6

**Published:** 2017-12-29

**Authors:** Brandi Leach, Perri Morgan, Justine Strand de Oliveira, Sharon Hull, Truls Østbye, Christine Everett

**Affiliations:** 1 Department of Community and Family Medicine, Duke University School of Medicine, Durham, NC USA; 2 Barts and The London School of Medicine and Dentistry, Queen Mary University of London, London, UK

**Keywords:** Primary care, Patient care team, Qualitative research

## Abstract

**Background:**

Current recommendations for strengthening the US healthcare system consider restructuring primary care into multidisciplinary teams as vital to improving quality and efficiency. Yet, approaches to the selection of team designs remain unclear. This project describes current primary care team designs, primary care professionals’ perceptions of ideal team designs, and perceived facilitating factors and barriers to implementing ideal team-based care.

**Methods:**

Qualitative study of 44 health care professionals at 6 primary care practices in North Carolina using focus group discussions and surveys. Data was analyzed using framework content analysis.

**Results:**

Practices used a variety of multidisciplinary team designs with the specific design being influenced by the social and policy context in which practices were embedded. Practices overwhelmingly located barriers to adopting ideal multidisciplinary teams as being outside of their individual practices and outside of their control. Participants viewed internal organizational contexts as the major facilitators of multidisciplinary primary care teams. The majority of practices described their ideal team design as including a social worker to meet the needs of socially complex patients.

**Conclusions:**

Primary care multidisciplinary team designs vary across practices, shaped in part by contextual factors perceived as barriers outside of the practices’ control. Facilitating factors within practices provide a culture of support to team members, but they are insufficient to overcome the perceived barriers. The common desire to add social workers to care teams reflects practices’ struggles to meet the complex demands of patients and external agencies. Government or organizational policies should avoid one-size-fits-all approaches to multidisciplinary care teams, and instead allow primary care practices to adapt to their specific contextual circumstances.

## Background

Robust primary care is known to be key to an efficient and effective health system [1, 2]. Compared to other economically developed nations, the US healthcare system places more focus on secondary and tertiary care than primary care. As a result, the US is top in healthcare spending, but at or near the bottom in health equity and quality outcomes [3]. While many policy makers, health delivery organizations, and funders would like to see a shift to more primary care delivery, a variety of factors, such as doctor shortages and an aging population with multiple chronic illnesses make strengthening a traditional primary care model increasingly difficult [4].

Current recommendations for strengthening the US healthcare system consider restructuring primary care into multidisciplinary teams as vital to improving quality and efficiency [5]. Primary care doctors alone are often unable to provide all needed care due to time constraints [6]. It is estimated that up to 47% of chronic care and 77% of preventive care could be delegated to other team members, potentially offsetting some demand for doctor services while improving access to care [7–9]. Evidence also suggests that well-organized multidisciplinary teams increase patient satisfaction [10, 11] and reduce doctor and staff burnout [12–14].

The recommendations, however, do little to recommend how to design these teams because there is a lack of information on the range of team designs being used [15]. Current approaches to distributing care among team members vary, resulting in a wide spectrum of team models [16, 17]. Team designs have expanded existing members' roles and added new members [15, 17–20]. For example, some practices have expanded the role of medical assistants (MAs) to include responsibility for preventive care management, patient education, and/or health coaching, and have demonstrated improvements in preventive service delivery and patient outcomes [21–24]. Other practices have included new members on the primary care team, such as physician assistants (PAs), nurse practitioners (NPs), pharmacists or behavioral health providers, to ensure access to needed services [25–28]. Many have reported encouraging results, suggesting that primary care practices have a range of team models from which to select.

Research offers some guiding principles for effective multidisciplinary care teams, including evidence from initial implementation. This work highlights the importance of factors such as physical spaces that facilitate interaction [29], cognitive factors that promote a sense of shared purpose and goals among team members [30, 31], effective communication [29, 30, 32], and leadership structures reflective of team goals and activities [29, 30]. Furthermore, this literature suggests that implementation of multidisciplinary teams should be undertaken with a flexible mindset that is sensitive to the needs of team members, as it may create unintended tensions, especially regarding role confusion [29].

Health systems outside the US have had varying degrees of success incorporating multidisciplinary teams into the delivery of primary care. For example, the uptake of multidisciplinary teams in Canada has been hampered by differences in funding and remuneration for primary care services across provinces, with some provinces adopting funding models that either explicitly or implicitly prioritize doctor-administered services [33, 34]. In contrast, the National Health Service in the UK embraced innovations conducive to multidisciplinary teams, such as funding support for non-doctor team members [35], a pay-for-performance system that encouraged the use of lower cost non-doctor personnel [36], and electronic health records that assist with care coordination [35], and as such has seen greater adoption of multidisciplinary teams than has occurred in the US.

The capacity of US primary care practices to create multidisciplinary primary care teams is complicated by a variety of issues. Firstly, due to a lack of healthcare workforce planning, the availability of healthcare professionals varies widely by geographic area [37, 38]. Also, reimbursement for services delivered can complicate team formation. Since the US has different payment structures for different populations [39], finding ways to consistently pay for the services of all team members can be challenging. A final complicating factor is that the US healthcare system is undergoing payment reform to value-based care, where quality of care delivered is factored into payment, but this reform has an uncertain future [40]. This uncertainty impedes efforts to identify and implement effective team designs. For example, in line with value-based payment reform efforts, many primary care practices are transitioning to patient-centered medical homes (PCMH). PCMH practices are characterized by assigned primary care providers for patients to facilitate continuity of care, coordinated care, enhanced access, and team-based care [41]. However the transition to a PCMH practice requires extensive administrative and financial resources that practices may be reluctant to commit without firm government backing for continued reform efforts.

As US primary care practices increasingly adopt team-based approaches to care, one issue that remains unclear is which models are being selected for implementation and which factors are influencing that selection. Primary care practices associated with large academic centers may have financial and workforce resources not available in rural areas [42–44]. The regulatory environment, reimbursement practices or organizational goals could influence and constrain the role of team members [16, 45, 46]. Additional factors related to payment or organizational policy could also be influencing the selection of team models.

The goal of this project was to understand approaches to the selection of primary care multidisciplinary team models in North Carolina (NC). The specific aims were: 1) to describe a range of primary care multidisciplinary teams being used in diverse communities in NC; 2) to describe how providers envision primary care practices’ ideal team designs; and 3) to identify perceived contextual facilitating factors and barriers to implementing those ideal teams.

## Methods

### Study design

This is a qualitative study using focus group discussions with additional data collected via questionnaires. Focus group discussions were conducted with 44 participants from six primary care practices in NC. Two survey instruments collected additional data on [1]: individual participants’ professional background and demographics, and [2] the characteristics of the practice and its patient population.

### Participants and recruitment

We used purposive sampling to select primary care practices, with the final sample size based on achievement of theme saturation. Practices were selected to represent a variety of geographic locations, population health characteristics, healthcare workforce characteristics, patient populations, and organizational structures. Primary care practices in NC with at least five people (clinical and administrative workers combined) were eligible for inclusion in our study. We used primary care practice information from the Duke Primary Care Research Consortium and the NC Medical Society Foundation’s Community Practitioner Program to recruit our sample. Practices were recruited by contacting practice managers via phone or email. Within each practice, with the assistance of practice personnel, we recruited 6–10 people from each practice to participate in the study. This included a mix of clinical and non-clinical personnel at each clinic, including doctors, physician assistants (PAs), nurse practitioners (NPs), clinical pharmacists, nurses, medical assistants (MAs), social workers, practice managers, or administrative staff.

### Interview structure

Focus group discussions occurred at the practices. To minimize potential concerns about discomfort to speak freely arising from staff hierarchies, we divided participants into two groups with doctors, PAs, NPs, pharmacists, and managers separate from nurses, medical assistants, social workers, and administrative staff. Focus groups were led by one of three study team members (CE, PM, or BL). The focus groups lasted approximately 45 min and were audiorecorded and transcribed. We used a set of key questions to guide the focus groups (Table 1) that allowed for follow-up questions to pursue emerging themes.

**Table 1:** Key questions from the focus group discussion guide

Question	
If a patient asked you, “What is the most important thing you do in this practice?” what would you tell them?	
How is your practice organized to deliver care?	
How did that practice structure come about?	
If you could create the ideal structure, what would it be?	
Do you have organized or designated teams as part of this practice?	

### Analysis

**Fig. 1:**
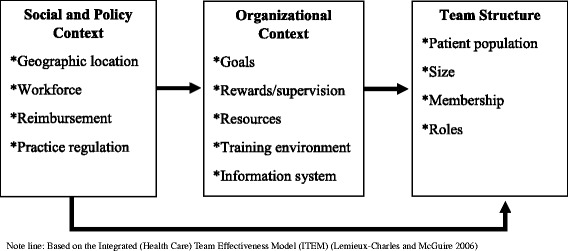
Conceptual model of primary care team design used for initial analytic framework

We evaluated the qualitative data using a framework content analysis approach [47]. Framework content analysis incorporates open, thematic coding into a theoretically-grounded analytic framework. Our initial framework (Fig. 1) was based on the Integrated (Health Care) Team Effectiveness Model (ITEM) [48]. This model posits that healthcare team structure is influenced not only by organizational context, but also by the larger social and policy context. Social factors influencing selection of team design may include geographic location (i.e., rural vs. urban), availability of workforce in different professions, and reimbursement policies. [46, 49, 50] Organizational factors such as goals, provider payment practices, and availability of resources might also impact the selection of team design [16, 45]. Finally, team structure is defined by the patient population served, the type and number of team members, and the roles of each member [48]. Based on the ITEM framework, our initial thematic coding categories captured the social and policy context, organizational context, and team structures. These were refined during the analysis to include categories addressing facilitators, barriers, and aspirations (see Table 2 for examples of comments coded under each category).

**Table 2:** Final thematic coding categories, with examples

Theme	Example
Team Design	
Structure	*It’s a free for all sister!*
Development	*We’re at a cross, it seems like, between hierarchy and collaboration. And trying to find middle ground.*
Aspirations	*I think certainly reducing some of the bureaucracy or the logistical complications of getting patients the care that in our medical decision-making they need to have.*
Organizational Context	
Barriers	*Financing. And space. Money and space. Cause right now, like we, if we had the money for a social worker, I wouldn’t have anywhere to put them.*
Facilitators	*Critical for me to have professional, capable, nursing staff and ancillary people to help me to make each patient visit the most efficient I possibly can.*
Culture	*You know, there’s two wars going on. Some providers are like, “I don’t want to see anymore [patients].” And then the administration is like, “But we gotta make money.”*
Values	*We really do try to put the patients first.*
Social & Policy Context	
Structure	*We have a lot of blueberry fields... so we have a lot of, they’re migrants, they go to Florida in Spring, they come here for the summer, they go to Michigan for the winter then turn right back around.*
Barriers	*You’re focused on getting a patient in and out and taking care of it in a, in a highly effective way … but all of a sudden it doesn’t matter cause they can’t afford it, or you know they’re living on the street or something like that.*
Facilitators	*There’s the Practice Transformation Network, you probably are familiar with that, is also driving some of our change. It’s not necessarily the impetus, but it’s helping give us some structure.*

Our analysis occurred in stages, beginning with an initial review of transcripts for familiarization and identification of key themes. This was followed by a secondary review to refine and adjust themes, and a systematic application of the refined themes to the full transcript data. The final stages reorganized and synthesized themed content for analysis and interpretation.

Qualitative analysis was led by two team members (CE and BL) who coded the transcripts independently before comparing and discussing their results and achieving consensus on any identified discrepancies in coding. The full study team provided feedback on preliminary results midway through data collection and after initial thematic coding and analysis. The full study team includes two doctors, three PA researchers, and one sociologist.

## Results

Our final sample included 44 healthcare professionals from 6 primary care practices in NC. Most participants were female (82%) and employed full-time at their clinic (90%). They had an average age of 45 years and had worked at the same clinic for an average of 8 years (Table 3). The practices in our sample encompassed a range of organizational structures situated within different contexts (Table 4). Five of the six practices employed between 1 and 2 doctors, as well as an additional 2 to 6 NPs or PAs. The sixth practice was associated with a large academic medical center and employed over 30 full and part-time doctors, including residents. Across the US, approximately 41% of primary care doctors in 2012 worked in practices with 1–2 doctors, a further 42% were in practices with 3–10 doctors, and 16% were in practices with 11 or more doctors [51].

**Table 3:** Participant characteristics, *N* = 44

Participant characteristics	
Age, in years [Mean/(SD)]	45/(11.1)
Time in current profession, in years [Mean/(SD)]	14/(9.0)
Time at current clinic, in years [Mean/(SD)]	8/(7.1)
Percent female	82%
Percent employed full-time	90%
*Number of each Professional Type Participating*	
Doctors	9
Nurse practitioners/Physician assistants	8
Pharmacists	1
Social workers	1
Nurses	5
Medical assistants	11
Administrative managers	4
Administrative staff	5

**Table 4:** Characteristics of clinics and their social context, *N* = 6

Clinic characteristics	Mean	SD	Min	Max
Median income of county^a^	$49,365	$10,322	$34,949	$67,309
Median income of city/town^a^	*	*	$19,719	$91,579
County health ranking (out of 100)^b^	35	33	96	1
County population size^a^	252,396	357,463	35,663	1,024,198
Estimated percent of patients with private insurance^c^	52%	27%	25%	91%
# practices that are patient-centered medical homes^d^	4			
# practices using electronic health records	5			

*Information not available due to small population size

^a^Source: US Census Bureau. Quick Facts, American Community Survey, 2015. http://www.census.gov/quickfacts. Accessed 2/14/17

^b^Note: County health rankings measure the health of counties in every US state using a population health model that includes policies and programs, health factors, and health outcomes. NC rankings are based out of 100 with 1 being the “healthiest” county. Source: Robert Wood Johnson Foundation, County Health Rankings & Roadmaps. 2016. http://www.countyhealthrankings.org/rankings/data/nc. Accessed 2/14/17

^c^Self-reported by practice managers. N = 4, 2 practices did not report insurance estimates

^d^For a description of patient-centered medical homes, please see: Devers KJ, Burton RA, Berenson RA. Will the patient-centered medical home transform the delivery of health care? 2011

### Team designs

**Fig. 2:**
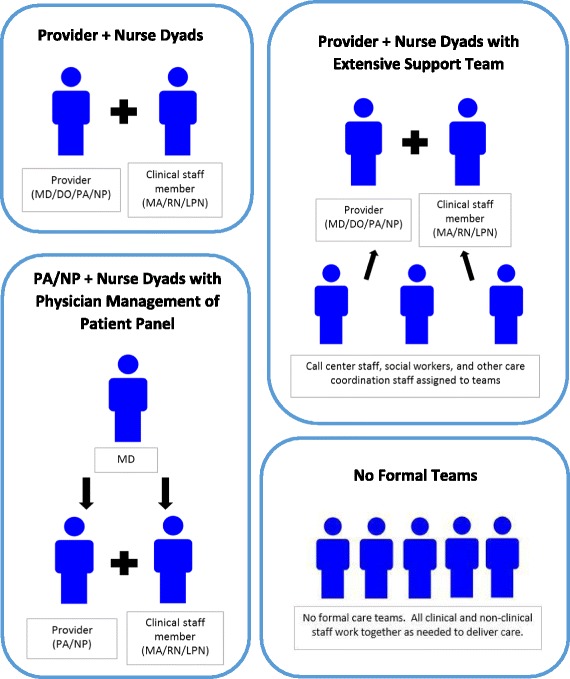
Team designs used by participating clinics, *N* = 6

The primary care practices used a variety of team designs, ranging from provider-nurse dyads (doctor, PA, or NP teamed with a nurse or medical assistant) to large multi-professional teams (Fig. 2). Most practices initially found it difficult to describe how their current team structures, staffing and workflow pattern came about, expressing a sentiment similar to that of one practice owner who said, “*it just sort of happened that way*.” When pressed, participants generally articulated that their current team structures were not the result of an explicit plan, but rather the result of various internal and external forces. Respondents from one suburban practice, for example, focused on hiring people with complementary skill sets and then developed their team structures around the individual strengths and preferences of their employees. As the practice owner described:

*“Each one of us individual provider[s], we provide…, cutting edge, really, medicine, but we're different, … what she's an expert on, I might not know much about. And vice versa.”*

In contrast, respondents from rural practices cited difficulties in hiring and retaining doctors due to workforce availability and resource limitations, leading them to rely on NP/PAs to provide a large portion of primary care services. One rural practice manager spoke of the difficulty of retaining *any* providers beyond the initial two-year contract period for which the providers receive educational loan forgiveness, given that experienced providers could make higher salaries elsewhere:

*“We get a lot of new NPs and PAs but as soon as they're trained they get snapped up by another provider … And we start all over again.”*

Practices facing resource limitations reported hiring staff to conform with insurance reimbursement practices that limit which provider types are eligible for incentive-based payments or can bill for specific services. For example, under the Medicaid program (government insurance for poor and disabled people) there are fewer restrictions on NPs to qualify for payments under the EHR incentive program than for PAs, which can make NPs a more economically desirable option [52]. These practices also used clinical staff (e.g. MAs) in expanded roles. Clinical staff generally appeared to value the additional responsibilities, but they also understood the underlying reasons for their expanded roles. As one MA from a rural practice stated:

*“With my paramedic background,…they brought me in cause I had the experience of the IVs and stuff, so basically I'm working as a … LPN, RN, but I get paid for MA.”*

In contrast, practices without significant resource limitations were able to emphasize non-financial factors when developing their teams. The approach described by members of a primary care practice associated with an academic medical center exemplified adaptive team development, with the practice adding new members or methods of coordination based on feedback from providers, staff and patients, the changing needs of the organization, or research findings. These decisions were not divorced from financial considerations, but in comparison to rural health practices and those working in higher poverty areas, this practice was able to develop its teams with more emphasis on meeting organizational goals, such as population health goals.

### Aspirations

Participants’ descriptions of ideal team designs frequently involved addressing gaps in important services. For example, four of the six practices cited the need for a social worker or care coordinator to manage complex patients. The specific reason for wanting a social worker/care coordinator varied by context with practices differentially emphasizing medical complexity, social complexity, or mental health needs based on contextual factors such as the characteristics of their patient population and availability of community resources. One nurse from a low-resource suburban practice captured the diversity of needs with her comment:

*“I think adding that mental health piece, because resources are so scarce, um, and maybe adding like some sort of case management services for our really complicated patients.”*

### Organizational context

Focus group participants identified numerous internal organizational aspects that affected the development and functioning of multidisciplinary teams. In general, organizational contexts were seen as facilitating multidisciplinary teams. Facilitating aspects of the organizational context included technological resources such as electronic communication and electronic health record (EHR) systems (“*Being able to leave messages for them in the EHR, that's helpful. That was a big step up from the paper charts*.” – administrative staff, rural practice); the skills and/or quality of their co-workers; organizational processes such as care team huddles [53], quality improvement initiatives, and staff training; and supportive and collaborative cultures within their practices (*“We’re a united front.” –* administrative staff, suburban practice).

Different organizational contexts, such as practice size and ownership structure, were associated with different perceived facilitating factors. For example, one independently-owned suburban practice described their freedom as a private practice to make organizational decisions and adopt innovative practices as facilitating their implementation of multidisciplinary teams. The practice owner described their freedom to act this way:

*“We're not really a part of any big corporate anything. … Before we make decisions…we try to really take into account everyone’s, how it will impact workflows on everyone.”*

Members of the practice associated with the academic medical center, in contrast, cited the extensive resources available to them as part of a large medical organization, including having a call center to help organize and triage incoming calls, and the ready availability of specialists who could be included on care teams, as facilitating the functioning of multidisciplinary teams.

Sometimes staff and providers would express differing views on whether something was a facilitating factor or a barrier, as in one practice without an EHR system. The owner of the practice resisted EHR implementation because of concerns over cost and additional time requirements that might limit the number of patients providers could see; whereas, the staff believed an EHR system would facilitate communication and improve their workflow.

Although perceived facilitating factors varied by organizational context, we found similarities that spanned contextual differences. Of particular note, members from every practice described their co-workers as essential for implementing multidisciplinary teams, with some emphasizing the skills and credentials of their co-workers and others highlighting their co-workers’ supportive, collaborative behavior. Among practices that mentioned using daily or weekly huddles, there was general agreement that huddles enhanced team functioning. As one nurse from a rural practice described it:

*“What has helped us a lot is a morning huddle…We'll have a better work flow ... That helps us to talk to each other and find out … what we're looking forward to during the day.”*

### Social and policy context

With only a few exceptions, the external social and policy context was viewed as a barrier to implementing well-functioning multidisciplinary teams in primary care practices by our focus group participants. The most commonly cited social and policy barriers to well-functioning multidisciplinary teams were socially complex patients, financial constraints, and policies of external agencies and parent organizations. The specific barriers cited varied by the practice’s context. For example, the perceived barriers mentioned by members of the practice associated with an academic medical center were largely related to factors controlled by their parent organization. These included the physical space of the practice which impeded team coordination, a belief among providers that the parent organization undervalued primary care, and hiring and staffing decisions. As expressed by one doctor:

*“The fact that the staff don't actually work for us, they work for [Parent Organization], you know it's very…we're not the final ones who say what they do. It's just kind of an odd, it's an odd setup.”*

In contrast, participants at privately owned practices described onerous insurance and government mandated requirements as impeding their delivery of quality team-based care:

*“The amount of things that we're required to do, increasing amount of things we're required to do. Checking boxes to the point you feel like you're not delivering care almost.” –* PA, suburban practice

*“We pay our employee and … half of their time's spent on the phone with the insurance company. So that's a major barrier.” –* Doctor, owner, suburban practice

Providers and staff at rural practices were likely to cite workforce availability as a barrier to implementing multidisciplinary teams, and participants at practices with high poverty patient populations reported financial barriers. One practice manager at a rural clinic with a high-poverty patient population expressed the view that delivering “*outstanding professional medical care*” often came into conflict with their financial circumstances. He stated that their practice’s goal was to recruit both doctors and PAs, because “*for the level of care and some of the complexity of our patients we'd love to have another [doctor] on staff*,” but the practice’s rural location and limited financial resources made it difficult to attract and retain doctors.

There were several government policies identified by study participants as facilitating their adoption of multidisciplinary teams. These included Medicaid payments for social workers, an improved state regulatory environment for clinical pharmacists, and the Centers for Medicare & Medicaid Services’ (CMS) Practice Transformation Networks which provide guidance and support to primary care practices transitioning to PCMHs or other quality-based patient-centered care approaches [54].

## Discussion

The six primary care practices in our study adopted an array of care team designs. Our research suggests that multidisciplinary team designs are influenced by the social and policy context in which practices are embedded, including factors such as the availability of financial and workforce resources, reimbursement practices, and patient complexity. Financial and workforce constraints appear to play a large role in determining multidisciplinary team designs for resource restricted practices. Within our study, workforce availability was especially important for multidisciplinary team design in rural practices, where a restricted doctor supply contributed to decisions to hire more NPs and PAs. Reimbursement practices influenced the types of professionals included on care teams as well as their roles. Limited resource practices used extended roles for MAs and other clinical staff in order to improve economic efficiency. Patient complexity also shaped care team design by influencing desired team members, such as a desire for social workers in practices with socially complex patients.

Primary care practices overwhelmingly located barriers to adopting their ideal multidisciplinary teams within the external social and policy context, and outside of their individual practices and outside of their control. In our study, these included parent organizations that controlled staffing decisions and undervalued primary care, complex patient populations requiring additional care coordination, and reimbursement policies that limited staffing choices. Their frustration with these external factors and their apparent inability to influence them were evident in the interviews.

Appropriate reimbursement policies for primary care services have been found by other researchers to be a key component in the success or failure of incorporating multidisciplinary team approaches to care outside the US [33, 35, 36]. Citing the examples of Canada [33, 35] and Australia [36], these researchers argue that even when there is political support for multidisciplinary teams, reimbursement models that continue to prioritize doctor-led services or offer poor or inconsistent funding streams for non-doctor team members hinder the uptake of multidisciplinary teams in practice. Funding models that offer more flexibility in service reimbursement, such as that found in the UK, may contribute to greater adoption of multidisciplinary teams in primary care [35].

In contrast to the perceived barriers, practice members viewed internal organizational contexts as the major facilitators of multidisciplinary primary care teams. These included factors facilitating communication (e.g. electronic communication and EHR systems, huddles), workforce characteristics (e.g. having skilled co-workers, availability of specialists), and practice cultures supportive of teamwork. External factors may also contribute to the adoption of multidisciplinary teams by providing financing for desired team members or offering guidance on practice transformation, but these were mentioned less often by our study sample.

Research on the effect of organizational factors on adoption of multidisciplinary teams in primary care highlights several important factors identified by our study. Most notably, this includes the importance of communication and team climate. Communication is argued to be both an important precursor to successful team implementation [30, 31] and an indicator of team functioning [29]. Research from England on the importance of team climate and culture has found conflicting results about its impact on patient outcomes [55, 56], but other research suggests that a positive team culture is necessary for team members’ satisfaction with their working environment [30, 57].

Despite differences, one consistent message from primary care practices is the desire for social workers and/or care coordinators on their teams. This finding echoes that of previous research which finds that care coordinators and social workers perform valued functions on care teams [58, 59]. Social workers offer an avenue for managing patient care plans, coordinating community resources, and providing clinical behavioral interventions [59]. As noted by the participants in our study, the main barrier to including social workers or care coordinators in multidisciplinary teams was a lack of funding.

### Strengths and limitations

A key strength of our interview-based approach is that it allows for the emergence of themes not previously identified by the researchers. This is important when studying emerging and developing areas such as team-based care. Our research is limited in that it only sampled practices from within a single state, although it captured a diverse set of contexts within that state and we achieved thematic saturation within our interviews. A larger sample would have permitted a more robust assessment of the effect of contextual factors on multidisciplinary care team functioning and design.

The composition of the study team presents a potential for bias in that all of the team members visiting practice sites were affiliated with a PA education program. This had the potential to affect comments made by participants regarding PAs. Although unable to eliminate this potential source of bias from within the interviews, the inclusion of several non-PA researchers on the larger study team ameliorated the potential for bias during the analysis.

Future research should seek to deepen our understanding of the range of multidisciplinary team models, and factors facilitating or impeding the establishment and ideal functioning of those models. This is needed to better inform practice transformation efforts and assist primary care practices with identifying a team model that will meet their specific needs and context.

## Conclusions

The successful implementation of multidisciplinary primary care teams is influenced not only by organizational context, but by the larger social and policy context. *External* factors are often perceived as barriers to implementing multidisciplinary teams, while facilitating factors largely originate from *within* clinics. And although the participants in this study tackled the challenges of team-based primary care with energy and creativity, the internal resources of most practices are not sufficient to overcome all of the externally-imposed barriers. Solutions could include flexible payment policies that allow for a variety of care team designs, supporting innovations in care delivery. Government or organizational policies should avoid one-size-fits-all approaches to multidisciplinary care teams, and instead allow primary care practices to adapt to their specific local and contextual circumstances.

## Abbreviations

CMS: Centers for Medicare and Medicaid Services
EHR: Electronic health records
LPN: Licensed practical nurse
MA: Medical assistant
NC: North Carolina
NP: Nurse practitioner
PA: Physician assistant
PCMH: Patient-centered medical home
RN: Registered nurse
